# Within-Subject Consistency of Unimodal and Bimodal Force Application during the Countermovement Jump

**DOI:** 10.3390/sports6040143

**Published:** 2018-11-08

**Authors:** Jason P. Lake, John J. McMahon

**Affiliations:** 1 Chichester Institute of Sport, University of Chichester, Chichester, West Sussex PO19 6PE, UK; 2 Directorate of Sport, Exercise and Physiotherapy, University of Salford, Frederick Road, Salford M6 6PU, UK; j.j.mcmahon@salford.ac.uk

**Keywords:** jump strategy, force platform, jump monitoring, temporal phase analysis

## Abstract

Countermovement jump (CMJ) force data are often time-normalized so researchers and practitioners can study the effect that sex, training status, and training intervention have on CMJ strategy: the so-called force–time curve shape. Data are often collected on an individual basis and then averaged across interest-groups. However, little is known about the agreement of the CMJ force–time curve shape within-subject, and this formed the aim of this study. Fifteen men performed 10 CMJs on in-ground force plates. The resulting force–time curves were plotted, with their shape categorized as exhibiting either a single peak (unimodal) or a double peak (bimodal). Percentage-agreement and the kappa-coefficient were used to assess within-subject agreement. Over two and three trials, 13% demonstrated a unimodal shape, 67% exhibited a bimodal shape, and 20% were inconsistent. When five trials were considered, the unimodal shape was not demonstrated consistently; 67% demonstrated a bimodal shape, and 33% were inconsistent. Over 10 trials, none demonstrated a unimodal shape, 60% demonstrated a bimodal shape, and 40% were inconsistent. The results of this study suggest that researchers and practitioners should ensure within-subject consistency before group averaging CMJ force–time data, to avoid errors.

## 1. Introduction

Counter-movement jump (CMJ) testing using a force platform is now routinely conducted across a variety of sporting-domains, in addition to many sports-science research studies [1,2]. This is because changes in CMJ strategy (i.e., the underpinning force and time characteristics before take-off) that either maintain or change jump height (JH) between testing occasions are thought to provide insight into neuromuscular function and fatigue [3,4]. Typically, so-called “gross” or “discrete” force–time variables (such as peak-force and peak-rate of force development) are reported following routine CMJ testing on a force platform. However, discrete force–time variables represent just one instantaneous force data point out of hundreds that are collected between jump initiation and take-off [1]. Consequently, these discrete variables do not explain how the CMJ force–time curves obtained for the entire jump (i.e., the combined unweighting, braking, and propulsion phases) change between testing occasions or differ between certain populations. An alternative approach, sometimes termed waveform analysis or Temporal Phase Analysis (TPA), involves time normalization of individual CMJ force–time curves (i.e., the expression of the time between jump initiation and take-off as a percentage rather than an absolute value), followed by a statistical comparison of the ensemble time-normalized force curves. This approach has gained popularity, with it being suggested that it overcomes the previously stated issues of discrete data analysis alone [1,3,5,6,7,8,9,10,11]. Thus, combining TPA and discrete methods can lead to a clearer understanding of biomechanical changes (or lack of) in CMJ strategy following certain training regimens, or differences between specific sporting cohorts. 

The first mention of TPA to study CMJ data appears to have been made by Cormie et al. [5], who hypothesized that it may be the effect that load has on the gradient of CMJ force, velocity, and power-time curves across the entire movement that underpins physiological responses to loaded jump-squat training. They found that external loading significantly affected the shape of the CMJ force–time curve, principally by increasing their magnitude but decreasing their slope. Subsequent work from this group and others went on to consider the effect of different training programs [6,11]. Additional applications of the TPA method include identifying how the shape of the CMJ force–time curve changes in response to neuromuscular fatigue [1,3] and differs between athletic populations [7,8,11]. Before deciding to utilize the TPA method, it is important to note that the pattern of force application in the CMJ can differ widely across subjects, with some demonstrating subtle differences (i.e., “v” or “u” shaped) during the unweighting phase [12], and many producing either a unimodal (one distinct force peak) or a bimodal (two separate distinct force peaks) force–time curve during the braking and/or propulsion phases [1,7,8,9]. Further sub-groups have been identified in studies of CMJ force–time characteristics based on subjects who achieved similar or different magnitudes of bimodal force peaks [13]. Because of such variation between subjects in CMJ strategies, it has been suggested that a single group TPA of CMJ force–time curves may mask performance-related factors that are affected by the “shape” of the force–time curve [9]. Sub-group analyses (e.g., creating unimodal or bimodal force–time curve subject sub-groups) enables clusters of force–time curves belonging to subjects who demonstrate similar CMJ force–time curve shapes and/or movement strategies to be created before any comparative analyses are conducted, which is suggested to be more effective when conducting a TPA [9].

A single-group analysis has been included in many cross-sectional studies involving a TPA of CMJ force–time curves [1,3,5,6,7,8,9,11,13,14], but this approach has recently been criticized [1] due to the reasons mentioned above. However, as previous work has only considered between-subject variation in unimodal and bimodal CMJ force–time curves [1], further research is required to establish whether similar variation is seen within-subject. Indeed, it is the monitoring of individual athlete’s CMJ force–time curve data that is of most interest to sports science practitioners. Furthermore, if subjects are found to produce different CMJ force–time curve shapes between trials (i.e., they switch between unimodal and bimodal patterns of force production), then this would have implications for the way in which sports-science researchers cluster individual time-normalized force–time curves together as part of the TPA process. Therefore, the aim of this study was to establish the within-subject agreement of the force–time curve shape exhibited during CMJ performance. Kennedy and Drake [1] explained that their subjects consistently demonstrated either a unimodal or bimodal force–time curve shape. Therefore, it was hypothesized that subjects in this study would consistently exhibit the same force–time curve shape. 

## 2. Materials and Methods

### 2.1. Subjects

Fifteen men (age: 21 ± 0.7 years, body mass: 84.3 ± 5.7 kg, height: 1.80 ± 0.56 m) who regularly participated in a variety of university-level sports (e.g., soccer, rugby (both codes), basketball and volleyball), volunteered to participate in this study and provided informed written consent. Subjects were excluded if they had suffered from a lower-body injury in the 6 months leading up to data collection. The study was approved by the institutional ethics committee and conformed to the principles of the World Medical Association’s Declaration of Helsinki.

### 2.2. Procedures

All subjects performed a standardized dynamic warm-up before all testing. This began with 2–3 min of upper and lower body dynamic stretching, using a previously described warm up [15]. Specifically, subjects performed 2 circuits of 10 repetitions each of “arm swings”, “lunge walk”, “walking knee lift”, “heel to toe lift” [16], and unloaded sub-maximal CMJs.

**Figure 1:**
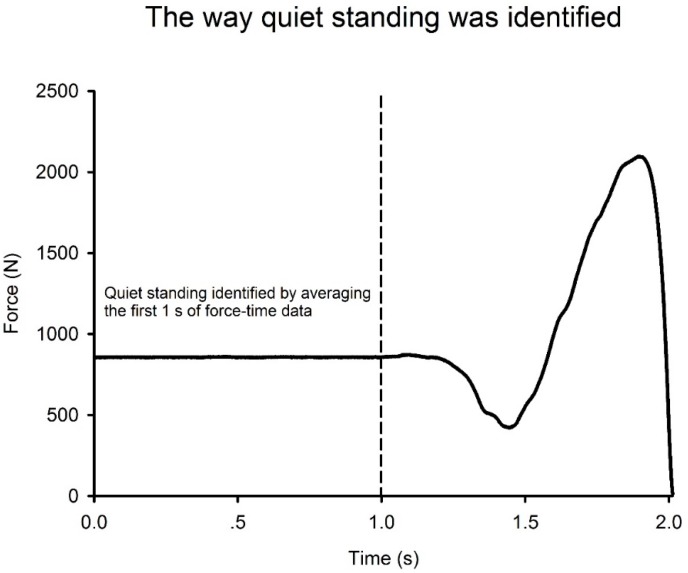
Representative force–time curves showing the different shapes and quiet standing period.

Subjects then performed 10 bilateral CMJs separated by 30 s of rest. To remove the impact of arm movement, subjects kept their hands on their hips throughout each jump. Before jump initiation subjects placed their hands on their hips and positioned each foot centrally on the corresponding force platform. Subjects were then instructed to stand perfectly still until given the words of command: “stand by, go!” The first word of command was issued 2 s after the subject had been instructed to stand perfectly still and indicated the start of data acquisition. There was a further gap of approximately 2 s between the words “stand by” and “go”. These instructions were to ensure that a sufficient period of quiet standing (Figure 1) was recorded [14,17]. Subjects were instructed to jump “as fast and as high as possible”. Jump performances were watched to ensure that subjects kept their hands on their hips throughout each jump. Trials were repeated if these criteria were not met.

### 2.3. Data Collection and Analysis

**Figure 2:**
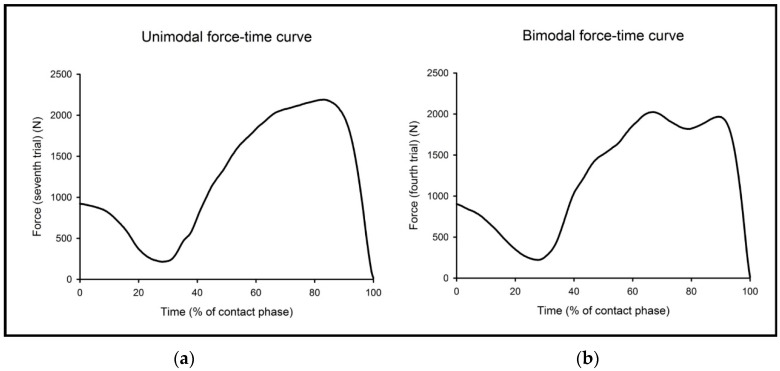
Examples of unimodal (**a**) and bimodal (**b**) force–time curves from the same subject.

Each CMJ was performed with feet placed on previously zeroed in-ground force plates (0.40 by 0.60 m Kistler Type 92538, Kistler Instruments, Hampshire, UK), that were positioned side-by-side and simultaneously recorded vertical force at 1000 Hz [17]. Left-side and right-side vertical forces were summed and presented graphically as single force–time curves for analysis. Force–time data analysis was done using a customizable Microsoft Excel spreadsheet. The force–time curves of each subject’s 10 CMJs were plotted, and after visual inspection of the force–time curve each trial’s shape was categorized as having either one distinct peak (unimodal) as shown in Figure 2a, or two distinct peaks (bimodal) as shown in Figure 2b.

### 2.4. Statistical Analysis

Within-subject agreement of the CMJ force–time curve shape was assessed by assigning a “0” when a unimodal force–time curve occurred and a “1” when a bimodal force–time curve occurred [18]. The total “0” and “1” were summed and the percentage agreement were calculated. Because of the difference in the amount of trials previously used for TPA, this was done over the first two trials [1], the first three trials [7,14], and the first five trials [3]. Additionally, to establish within-subject consistency over more prolonged testing protocols this process was also applied to all 10 trials. However, because a certain amount of this agreement is likely to have occurred by chance [19,20], the kappa coefficient was used to assess agreement. The kappa coefficient describes the proportion of agreement between measures after any agreement by chance has been removed [19]. The kappa coefficient was calculated in SPSS v.23 (SPSS Inc., Armonk, NY, USA), while its 95% confidence intervals were calculated in a Microsoft Excel spreadsheet using methods published in the literature [19]. The agreement scale presented by Viera and Garrett [20] was used to quantify agreement, where kappa values of 0.01–0.20, 0.21–0.40, 0.41–0.60, 0.61–0.80, and 0.81–0.99 represented slight, fair, moderate, substantial, and almost perfect agreement respectively.

## 3. Results

The force–time curve shapes that each subject demonstrated across the ten trials are presented in Table 1. It is worth noting that those who did not demonstrate a consistent force–time curve shape did not present any discernible pattern in the shape of their force–time curve. When two trials were considered, 13% of subjects consistently demonstrated a unimodal force–time curve shape, while 67% of subjects consistently demonstrated a bimodal force–time curve shape. The remaining 20% of subjects did not consistently demonstrate the same force–time curve shape. The kappa analysis showed very poor within-subject agreement (kappa [±95% confidence interval] = −0.023 [−0.206, 0.160]).

When three trials were considered, the same pattern was found; 13% of subjects consistently demonstrated a unimodal force–time curve shape, while 67% of subjects consistently demonstrated a bimodal force–time curve shape. The remaining 20% of subjects did not consistently demonstrate the same force–time curve shape. The kappa analysis showed very poor within-subject agreement (kappa [±95% confidence interval] = −0.216 [−0.368, −0.064]).

When five trials were considered, none of the subjects consistently demonstrated a unimodal force–time curve shape, but 67% of subjects consistently demonstrated a bimodal force–time curve shape. The remaining 33% of subjects did not consistently demonstrate the same force–time curve shape. The kappa analysis showed very poor within-subject agreement (kappa [±95% confidence interval] = −0.027 [−0.049, −0.005]).

Finally, when ten trials were considered, none of the subjects consistently demonstrated a unimodal force–time curve shape, but 60% of the subjects demonstrated a bimodal force–time curve shape. The remaining 40% of subjects did not consistently demonstrate the same force–time curve shape. The kappa analysis showed very poor within-subject agreement (kappa [±95% confidence interval] = 0.063 [0.006, 0.120]). 

## 4. Discussion

The aim of this study was to establish the within-subject agreement of the force–time curve shape exhibited during CMJ performance, and it was hypothesized that subjects would consistently exhibit the same force–time curve shape. The results did not support this hypothesis and therefore do not agree with results recently presented in the literature [1]. The kappa coefficients failed to reach acceptable agreement when two, three, five or 10 CMJ trials were considered. This finding has important implications for the way researchers and practitioners who use force plates to assess CMJ performance study the CMJ force–time curve data in the future. This is because it shows that grouping individual time-normalized force curves together could lead to the misrepresentation of individual athlete CMJ strategies and therefore mislead the interpretation of training results and subsequent training prescription. It also brings into question conclusions that have been drawn from previous research in this field [1,3,5,6,7,8,9,13,14].

Kennedy and Drake [1] suggested that their subjects consistently adopted either a unimodal (~48%) or a bimodal (~52%) force–time curve pattern between the two CMJ trials that they performed in total. In the present study, 20% of subjects varied between producing a unimodal or bimodal CMJ force–time curve when their first two trials were considered, and this increased to 40% across the full 10 CMJ trials recorded. The reason for differences between studies, when considering the first two trials alone, might be due to the present study including subjects who competed in a range of sports, as training background has been previously shown to influence the shape of the CMJ force–time curve [6,7,8]. McMaster et al. [13] also found within-subject differences in unimodal and bimodal (including demonstration of bimodal equal peaks and bimodal high-low (force) peaks) CMJ force–time curves across the six trials that they performed. This was in agreement with the results of the present study, despite all subjects of their own study being professional rugby-union players. McMaster et al. [13] presented these results for three individual subjects only, thus it is unknown how many of the 21 subjects tested in the study also showed between-trial variability in their pattern of force application. When the ensemble average CMJ force–time curves for each subject were considered by McMaster et al. [13], ~29%, ~38%, and ~33% of subjects produced unimodal, bimodal high-low peaks and bimodal similar peaks respectively. This shows that a bimodal pattern of CMJ force application seems to be the most common amongst subjects, which is in line with the present study. 

Professional rugby league players perform CMJ with a higher reactive strength-index modified, by jumping higher and with a shorter time to take-off, demonstrating less bimodal force–time curve shapes compared to those who achieved a lower reactive strength-index modified [6,7]. Conversely, a recent study by Kennedy and Drake [1] showed that neither jump height or reactive strength-index modified differed substantially between rugby union players who demonstrated a unimodal or bimodal CMJ force–time curve. However, the latter study did suggest that a bimodal CMJ force–time curve may represent inefficient use of the stretch-shortening cycle. Therefore, owing to the large between-subject variability in braking center-of-mass displacement (i.e., the bimodal group showing larger braking center-of-mass displacement and longer time to take-off), the TPA method should be avoided in cross-sectional (between-subject) studies. McMaster [13] also reported a shorter time to take-off and a higher flight-time to contraction-time ratio (which relates almost perfectly to reactive strength-index modified [21]) for the unimodal CMJ force–time curve group.

Findings presented by McMaster et al. [13] suggest that jumpers who exhibit a unimodal force–time curve shape perform their CMJ faster (less time from jump initiation to take-off), but this appears to result in them not being able to generate and apply an impulse sufficient to “out-jump” those who exhibit the bimodal force–time curve shape. This suggests a trade-off between performing the movement as quickly as possible and jumping as high as possible. It should also be noted that performing the jumps faster may not decrease time to take-off (and vice versa). Unfortunately, it is not known whether instructions were given to the subjects in this study, so it is difficult to be certain. This will need to be considered in future research. Another potential limitation to some of the research in this area is that relatively low sampling-frequencies have been used (200 Hz rather than 1000 Hz) [3,13]. Clearly, reduced signal resolution may influence our ability to interpret the content of force–time data. However, further research is needed to establish whether sampling-frequency influences CMJ force–time curve shape.

Though the results of this study have produced some clear findings, this study is not without its limitations. Firstly, we used a relatively small sample size, and the fact that subjects participated in a variety of university-level sports may have further confounded our findings. Therefore, future research into the agreement of within-subject force–time curve shape may benefit from recruiting a larger sample size to enable stratification according to sport and CMJ performance level [1,6,8]. Secondly, the instruction to “jump as high and fast as possible” that was given to our subjects may have influenced performance. Kennedy and Drake [1] suggest that the shape of the force–time curve may provide an opportunity to change the instructions we give to subjects to help maximize performance, although confirmation requires further research. For example, practitioners working towards minimizing CMJ performance-time may elicit a unimodal force–time curve shape from their athletes. Conversely, practitioners working towards maximizing jump height may elicit a bimodal force–time curve shape. Further research is needed to establish if this is true. Thirdly, the plots of all the force–time curves produced by the 15 subjects were visually inspected and categorized subjectively. There are currently no accepted criteria to guide researchers and practitioners on how to more objectively quantify and categorize the shape of CMJ force–time curves. Therefore, this should be considered when interpreting the results of this study. Finally, we cannot rule out the possibility that successive fatigue underpinned changes in the force–time curve shapes that were demonstrated by our subjects.

## 5. Conclusions

The results did not support our hypothesis that subjects would consistently exhibit either unimodal or bimodal CMJ force–time curves. Furthermore, kappa-coefficients failed to reach acceptable agreement when two, three, five, or 10 CMJ trials were considered. This finding has important implications for the way researchers and practitioners’ study CMJ force–time curve data in the future, as it shows that grouping individual time-normalized force curves together could lead to the misrepresentation of individual athlete CMJ strategies, and therefore mislead the interpretation of training results and subsequent training prescription. We therefore recommend that researchers and practitioners consider data on an individual basis when using this method to gauge athlete CMJ strategy, training status, and the results of training interventions.

## Figures and Tables

**Table 1:** The force–time curve shapes that each subject demonstrated across the ten trials.

Subject	Trials									
	1	2	3	4	5	6	7	8	9	10
1	Bi	Bi	Bi	Bi	Bi	Bi	Bi	Bi	Bi	Bi
2	Uni	Uni	Uni	Bi	Uni	Uni	Bi	Bi	Bi	Bi
3	Bi	Bi	Bi	Bi	Bi	Bi	Bi	Bi	Bi	Bi
4	Bi	Bi	Bi	Bi	Bi	Bi	Bi	Bi	Bi	Bi
5	Uni	Bi	Bi	Bi	Uni	Uni	Bi	Uni	Bi	Bi
6	Bi	Bi	Bi	Bi	Bi	Bi	Bi	Bi	Bi	Bi
7	Bi	Uni	Bi	Bi	Bi	Bi	Bi	Bi	Bi	Bi
8	Bi	Bi	Bi	Bi	Bi	Bi	Bi	Bi	Bi	Bi
9	Uni	Bi	Bi	Bi	Bi	Bi	Bi	Bi	Bi	Bi
10	Bi	Bi	Bi	Bi	Bi	Uni	Uni	Uni	Bi	Uni
11	Bi	Bi	Bi	Bi	Bi	Bi	Bi	Bi	Bi	Bi
12	Bi	Bi	Bi	Bi	Bi	Bi	Bi	Bi	Bi	Bi
13	Bi	Bi	Bi	Bi	Bi	Bi	Bi	Bi	Bi	Bi
14	Uni	Uni	Uni	Bi	Bi	Bi	Bi	Bi	Bi	Bi
15	Bi	Bi	Bi	Bi	Bi	Bi	Bi	Bi	Bi	Bi

* Uni = unimodal force–time curve shape demonstrated; Bi = bimodal force–time curve shape demonstrated.
